# mmnet: An R Package for Metagenomics Systems Biology Analysis

**DOI:** 10.1155/2015/167249

**Published:** 2015-09-03

**Authors:** Yang Cao, Xiaofei Zheng, Fei Li, Xiaochen Bo

**Affiliations:** ^1^Department of Biotechnology, Beijing Institute of Radiation Medicine, 27 Taiping Road, Haidian District, Beijing 100850, China; ^2^Department of Biochemistry and Molecular Biology, Beijing Institute of Radical Medicine, 27 Taiping Road, Haidian District, Beijing 100850, China

## Abstract

The human microbiome plays important roles in human health and disease. Previous microbiome studies focused mainly on single pure species function and overlooked the interactions in the complex communities on system-level. A metagenomic approach introduced recently integrates metagenomic data with community-level metabolic network modeling, but no comprehensive tool was available for such kind of approaches. To facilitate these kinds of studies, we developed an R package, *mmnet*, to implement community-level metabolic network reconstruction. The package also implements a set of functions for automatic analysis pipeline construction including functional annotation of metagenomic reads, abundance estimation of enzymatic genes, community-level metabolic network reconstruction, and integrated network analysis. The result can be represented in an intuitive way and sent to Cytoscape for further exploration. The package has substantial potentials in metagenomic studies that focus on identifying system-level variations of human microbiome associated with disease.

## 1. Introduction

The human microbiome has been proved to play a key role in human health and disease. Various microorganisms species along with wide range of interactions among them structure the microbial communities as inherently complex ecosystems across different human body sites, such as gut, oral cavity, and skin [1, 2]. The microbes in our bodies are crucial for human life, having profound influence on human physiology and health [3–5]. Most of these microbes are still unculturable and uncharacterized [6]. Furthermore, dynamic shifts in microbial community structure will change the immunity and metabolic function and cause various diseases, including obesity, inflammatory bowel disease (IBD), and Crohn's disease [7–9].

Traditional culture-dependent methods are restricted by the small number of cultured species and often fail to describe the less abundant species [10]. To address this challenge, metagenomics (defined as environmental and community genomics), a culture-independent technology, has been developed and widely used for microbial community analysis [11]. Specifically, unlike the initial capillary sequence-based or PCR-based metagenomic approaches, high-throughput metagenomic approaches based on next generation sequencing (NGS) make metagenomic analysis more sensitive, broader, and cheaper, providing critical insights into microbe-host interaction in large scale [12, 13].

A key challenge of applying metagenomics to microbial community is metabolic network reconstruction from metagenomic data. Previous studies focused mainly on the “parts list” of the microbiome and overlooked the interactions in the complex communities on system-level [14, 15]. However, an integrated approach to reconstruct the metabolic network by integrating metagenomic data with genome-scale metabolic modeling was introduced recently [7]. This metagenomic system biology method serves the entire microbiome as a single superorganism and utilizes the computational systems biology and complex network theory, providing comprehensive systems-level understanding of the microbiome by integrating metagenomic data with genome-scale metabolic modeling. However, there is no comprehensive tool available for such kind of approaches. Here, we present an open source R package named* mmnet* to implement community-level metabolic network reconstruction. Moreover,* mmnet* implements a set of functions to construct an automatic pipeline from functional annotation of metagenomic reads to integrated network analysis. The result can be represented in an intuitive way and sent to Cytoscape for further exploration. The source code is published under GNU GPLv2 license and is freely available on the Bioconductor project (http://www.bioconductor.org/packages/devel/bioc/html/mmnet.html).

## 2. Methods and Implementation

### 2.1. Methods

*Metagenomic Sequence Reads Annotation.* To assess the functional capacity of microbial community, metagenomic sequence reads need alignment to a database of known genes and can be achieved with several well-characterized functional databases including KEGG orthology (KO) [16] and COGs [17]. The MG-RAST [18], a stable, extensible, online metagenomics analysis platform, is well maintained and provides a RESTful web API (Application Programming Interface) http://api.metagenomics.anl.gov/api.html. In this package, we annotate metagenomic reads by calling the RESTful web API of MG-RAST with R package* Rcurl*. If the metagenomic sequences have been annotated on MG-RAST, it will return the corresponding metagenome ID that already exists in MG-RAST without duplicated annotation.

*Enzymatic Gene Abundances Estimation.* M5NR [19], MG-RAST uses to annotate sequences, is an integration of many sequence databases into one single comprehensive, searchable database including most common functional databases like KO, EBI, and SEED. Once metagenomic sequences were annotated, KO information was filtered and extracted in the annotation profile from MG-RAST for subsequent metabolic network reconstruction. Enzymatic genes abundances can be estimated according to the following guidelines. (1) The count for the sequence matched a single reference sequence that had been annotated with more than one KO split evenly between all the KO annotations. (2) The count for the sequence matched more than one KO-annotated reference sequence with the same *e*-value split evenly between KOs of all matched reference sequences. Relative abundances of enzymatic genes in each sample can be finally computed by normalizing the counts of the reads for each KO with the total number of reads of enzymatic genes accounting for the different sample depths.

*System-Level Metabolic Network Construction.* A system-level metabolic network was constructed from the entire enzymatic genes (KO) in any sample. Reference metabolic data used to construct metabolic network, which consists of KO id and metabolic reactions, was obtained by phasing KEGG metabolic pathway dataset using KEGG REST API. Each enzyme may be associated with multiple reactions, and each reaction may be associated with multiple enzymes. In this metabolic network, enzymes are connected with directed edges, and a directed edge from enzyme A to enzyme B indicates that a product metabolite of a reaction catalyzed by enzyme A is a substrate metabolite of a reaction catalyzed by enzyme B.

To examine whether enzymes that are associated with a specific host state exhibit some topological features in the SSN, we calculated most common topological properties (Table 1) of each node in the network.

*Metabolic Network Analysis.* Comparing the abundances of enzymatic genes in different samples (e.g., disease and healthy samples) can reveal enzymes associated with specific host state. The package mmnet implements three methods to measure the differential abundance between different samples, including odds ratio (OR), RANK, and Jensen-Shannon Diverge (JSD). First, OR was calculated according to

(1) $$\begin{array}{c} \text{OR}\left(k\right)=\frac{\left[\sum_{s={\text{state}}_{1}}^{}A_{sk}/\sum_{s={\text{state}}_{1}}^{}\left(\sum_{i\neq k}^{}A_{si}\right)\right]}{\left[\sum_{s={\text{state}}_{2}}^{}A_{sk}/\sum_{s={\text{state}}_{2}}^{}\left(\sum_{i\neq k}^{}A_{si}\right)\right]}, \end{array}$$

where *A*_*sk*_ represents the abundance of enzyme *k* in sample *s* and state_1_ and state_2_ represent two types of samples with different state. The differential abundance score was defined as the absolute value of the fold change in OR, abs[log_2_⁡(OR)]. Once method RANK was selected, the enzyme abundances were ranked within each sample from most abundant to least abundant first. The difference abundance score was then measured with the difference between the mean ranks of samples in different state. Finally, users can examine the divergence using the Jensen-Shannon divergence algorithm to quantify the differential abundance score between samples in different state.

### 2.2. Implementation

**Figure 1:**
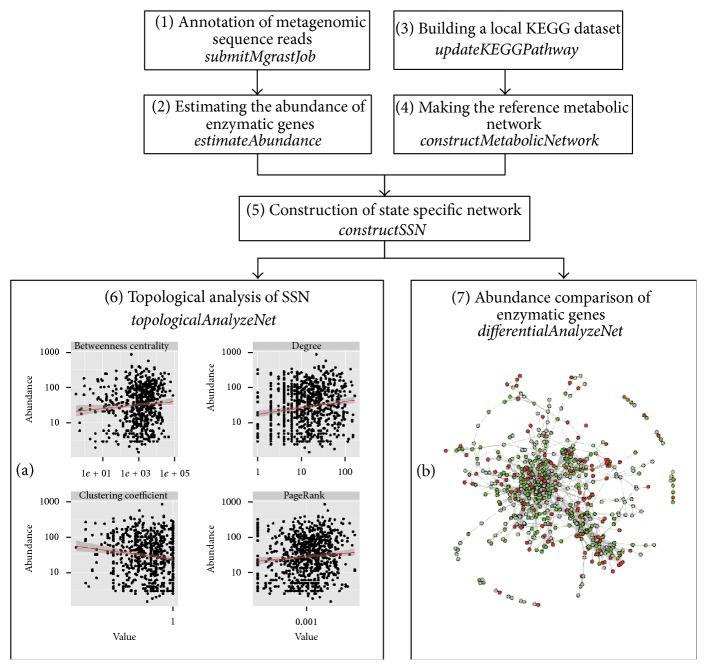
A typical analysis pipeline supported by the mmnet package.

A typical analysis pipeline for metagenomic systems biology supported by this package (Figure 1) starts with functional annotation and abundance estimation, then state specific network (SSN) construction, and finally topological and differential network analysis.* mmnet* is released as an R package including seven main functions (Figure 1):* constructSSN, submitMgrastJob, estimateAbundance, updateKEGGPathway, constructMetabolicNetwork, topologicalAnalyzeNet, and differentialAnalyzeNet*. All of these functions will be introduced as follows.

#### 2.2.1. Metagenomic Sequence Reads Annotation

*submitMgrastJob* is used for metagenomic sequence reads annotation on MG-RAST. Before sequence annotation, users should log in into MG-RAST first. Acceptable sequence data can be in FASTA, FASTQ, or SFF format. A MG-RAST ID represents that the annotation data is returned by this function. Then annotation profile will be obtained using function* getMgrastAnnotation* which takes the MG-RAT ID as input. The output is a data-frame in which one row corresponds to one KO annotation information.

#### 2.2.2. Estimating the Abundance of Enzymatic Genes

*estimateAbundance* is to estimate the abundances of enzymatic genes in the annotation profile mentioned above. As a result, a Biological Observation Matrix (BIOM) [20] file format, which is designed to be a general-use format for representing biological sample by observation contingency tables, is returned to encode enzymatic gene abundance profiles.

#### 2.2.3. Building a Local KEGG Dataset

*updateKEGGPathway* is used to build or update a local KEGG metabolic dataset for improving the efficiency of analysis and avoiding frequently repeated data downloads. This function is provided in* mmnet* package for users to build and update a local version of KEGG metabolic pathway dataset. The local metabolic data named* RefDbcache* is saved in the “∖*.mmnet*” subdirectory under user-specified folder, default under the user's home directory.

#### 2.2.4. Making the Reference Metabolic Network

*constructMetabolicNetwork* is used for construction of the reference metabolic network based on the metabolic reaction relationships in KEGG metabolic pathway dataset. A prebuilt reference metabolic network of class* igraph* has been integrated in* mmnet* package, so that the rebuilding of the network is unnecessary unless there are updates on the KEGG metabolic pathways.

#### 2.2.5. Construction of STATE Specific Networks

*constructSSN* is designed to construct a SSN by calling function* constructMetabolicNetwork*. Its input is an abundance profile of a sample and the output is subnetworks of the reference network composed of only the enzymatic genes identified from samples in a given biological state. This network is also of class* igraph*. In addition, the abundances of enzymatic genes are taken as node attributes in the SSN. The resulting SSNs can be seamlessly analyzed in R environment by built-in functions or in Cytoscape [21] by utilizing RCytoscape package [22].

#### 2.2.6. Topological Analysis of SSN

*topologicalAnalyzeNet* is to compute and illustrate the correlations between the topological properties of enzymatic genes and their abundances (Figure 1(a)). The input of the function is a single SSN. The output is also an* igraph*, in which all the calculated topological properties of nodes in SSN are stored as node's attributes and can be exported for further analysis.

#### 2.2.7. Differential Analysis of SSN

*Differential AnalyzeNet* is to calculate the differential abundance score of metabolic networks with different states. The function takes a list of SSNs as input and outputs a community-level metabolic network in which the significantly enriched or depleted enzymes are marked by colors (Figure 1(b)).

More detailed description of these functions and the package instructions is referred to in reference manual http://www.bioconductor.org/packages/devel/bioc/vignettes/mmnet/inst/doc/mmnet.pdf.

## 3. Results

To illustrate the analysis pipeline made by this package, we use a part of the public dataset containing 18 microbiomes from 6 obese and lean monozygotic twin pairs and their mothers [8]. 454 FLX pyrosequencer was used to carry out deep metagenomic shotgun sequencing of total fecal community DNA of 18 obese or lean samples. These metagenomic sequences have been annotated on MG-RAST, and the annotated data is available in MG-RAST. Thus, we downloaded the annotation profiles of these samples using* getMgrastAnnotation* directly without submitting a MG-RAST job. Apparently, users can annotate the sequenced metagenomic reads using function* submitMgrastJob* manually. For example, one of twin pairs (mgm4440616.3 and mgm4440824.3) and the function annotations can be accessed as follows:

 
source(" http://bioconductor.org/biocLite.R ")
 
biocLite("mmnet")
 
library(mmnet)
 
pid <- c("4440616.3","4440824.3")
 
names(pid) <- c("obese", "lean")
 
annot <- lapply(pid,
 
getMgrastAnnotation)

The relative abundances of enzymatic genes in the two samples were estimated from the functional annotations, and then the corresponding SSNs were built. For these two samples, 1345 KOs were identified in total. The correlation coefficient tested with Pearson's method is 0.92, which indicated that the relative enzymatic gene abundance across these two samples was highly concordant:

 
abund <- estimateAbundance(annot)
 
ssn <- constructSSN(abund)

Based on the SSNs, we intuitively explored the correlations between the topological features of enzyme in the SSN and their abundance (Figure 1(a)) and performed differential network analysis to identify potential enzymes associated with obese (Figure 1(b)). After differential metabolic network analysis, enzymatic genes that are associated with specific state appear as colored nodes (red = enriched and green = depleted):

 
lapply(ssn, topologicalAnalyzeNet)
 
differentialAnalyzeNet(ssn, sample.
 
state = names(pid))

Notably, only two samples were taken for testing* mmnet* accounting for saving computing resources and time; the results will be more meaningful when more samples were tooken for analysis.

## 4. Conclusions

The metagenomic approach on metabolic network provides a system-level understanding of the microbiome and gains insight into variation in metabolic capacity. It is very useful for studying the metabolic activity and specifically complex inherent interactions by serving the microbial community as a single supraorganism. In this paper, we present the* mmnet* package to support metagenomic network reconstruction as an integrated way in R environment and to build automatic pipelines running from metagenomic sequencing reads to community-level metabolic network. This package has substantial potentials for community metabolism analysis.

## Figures and Tables

**Table 1:** The topological properties calculated in the SSN.

Topological features	Description
Betweenness centrality	The fraction of shortest paths between node pairs that pass through the node

Clustering coefficient	The number of triangles (3 loops) that pass through this node

PageRank	The number and PageRank metric of all nodes that link to the node

Degree	The number of edges connected to the node

## Availability and Requirements

project name: mmnet;
project home page: http://www.bioconductor.org/packages/devel/bioc/html/mmnet.html­;
operating system(s): platform independent;
programming language: R;
other requirements: R 3.1.0 or higher;
license: GNU GPLv2;
any restrictions to use by nonacademics: none.
